# Isolated Tachycardia Presenting After Pfizer-BioNTech COVID-19 Vaccination

**DOI:** 10.7759/cureus.16706

**Published:** 2021-07-28

**Authors:** Charles Tate, Luay Demashkieh, Wael Hakmeh

**Affiliations:** 1 Emergency Medicine, Chicago Medical School at Rosalind Franklin University of Medicine and Science, North Chicago, USA; 2 Emergency Medicine, St. George's University School of Medicine, Grenada, GRD; 3 Emergency Medicine, Western Michigan University Homer Stryker M.D. School of Medicine, Kalamazoo, USA

**Keywords:** covid-19 vaccine, sinus tachycardia, adverse reaction from vaccination, post-vaccination, pfizer-biontech

## Abstract

A 29-year-old woman presented to the emergency department with palpitations and a heart rate of over 140 beats per minute that started approximately six to eight hours after administration of her second COVID-19 vaccination. Many side effects have been associated with the administration of vaccines. We present the first documented case of tachycardia and palpitations, in the absence of other signs or symptoms, presenting within hours of receiving the Pfizer-BioNTech COVID-19 vaccination. Clinicians should be aware that this appears to be benign and resolved within 24 hours in our patient.

## Introduction

Tachycardia has a long differential. Adverse reactions from medications and vaccines are common. Tachycardia, in the absence of other symptoms, has not been previously described as a side effect of the Pfizer-BioNTech COVID-19 mRNA (BNT162b2) vaccine. We have conformed to local regulations on reporting side effects of drugs and reported it to local authorities and the manufacturer (report #fd91cf6a-2fc1-4929-8844-b74652bc2b69).

## Case presentation

A 29-year-old woman presented with palpitations that started approximately six to eight hours after administration of her second Pfizer-BioNTech COVID vaccination. Her initial vital signs were temperature 37.4˚C, blood pressure 152/99, heart rate 135, and 100% oxygen saturation. She denied any chest pain, dyspnea, vomiting or diarrhea, cough, abdominal pain, genitourinary symptoms, rash, or travel. There was no bleeding, dark or tarry stools, or change in menstruation. There was no alcohol use to raise concern for possible withdrawal. She was not under stress, did not drink a significant amount of caffeine, denied using any drugs or taking any over the counter medications (including herbals or supplements), and had no other symptoms to suggest another etiology, such as an infectious process. A complete blood count (CBC), basic metabolic panel (BMP), thyroid-stimulating hormone (TSH), free thyroxine (free T4), D-dimer, and two sets of high-sensitivity troponin were all within normal limits (Table 1). Electrocardiography (ECG) showed sinus tachycardia with no ST or T-wave abnormalities. Pregnancy test, urinalysis, and urine drug screen were negative. Computed tomography of the chest showed no evidence of pulmonary embolism or any identifiable pathology. The patient was afebrile and hemodynamically stable, except for the tachycardia (heart rate (HR): 130s). Three liters of lactated ringers were administered, even though the patient did not appear dehydrated. Ativan (lorazepam) and Tylenol were also administered. Despite all these measures, the tachycardia persisted. The patient was given a dose of metoprolol orally and discharged home. On outpatient follow-up, patient’s tachycardia had resolved by the following day, and on subsequent follow-up, the tachycardia did not recur.

**Table 1:** Laboratory Studies BUN: blood urea nitrogen; FEU: fibrinogen equivalent units; RBC: red blood cells; T4: thyroxine; TSH: thyroid-stimulating hormone; WBC: white blood cells

	Value	Normal Range
WBC	7.9	4 - 11 x 10^9^/L
Hemoglobin	12.1	12 - 16 g/dL
Hematocrit	36.5	36% - 47%
Platelet	252	140 - 440 10^9^/L
Glucose	106	70 - 99 mg/dL
Sodium	138	135 - 145 mmol/L
Potassium	4.3	3.5 - 5.3 mmol/L
Chloride	107	98 - 108 mmol/L
Bicarbonate	22	23 - 32 mmol/L
BUN	10	6 - 20 mg/dL
Creatinine	1	0.6 - 1.1 mg/dL
Calcium	8.6	8.6 - 10.3 mg/dL
D-dimer	0.41	< 0.5 µg/mL (FEU)
Troponin (5th gen)	< 6	< 14 ng/L
Troponin after 2 hrs	< 6	< 14 ng/L
TSH	0.24	0.27 - 4.20 (iU)/mL
Free T4	1.5	0.8 - 1.7 ng/dL
Pregnancy test	Negative	
Urinalysis	No RBC, WBC or bacteria	
Drug screen	Negative	

## Discussion

Tachycardia has been twice previously described as a side effect of the Pfizer-BioNTech COVID-19 vaccine [1-2]. Garcia et al. reported on three physicians who received this vaccine and developed tachycardia in the absence of fever after immunization [1]. In this case series, the tachycardia occurred within 24 hours after immunization and lasted a total of 16 hours, similar to our case. All three physicians had previously tested positive for COVID-19. Reddy et al. described a patient developing a new-onset postural orthostatic tachycardia syndrome which occurred after the administration of the Pfizer-BioNTech COVID-19 vaccine [2]. Unlike these patients who experienced a multitude of signs and symptoms, our patient experienced only isolated tachycardia. The mechanism for tachycardia in our patient remains unclear.

Vaccines can occasionally cause tachycardia. Park et al. reported on a 5-month-old child who developed paroxysmal supraventricular tachycardia within four to six hours after each of the two, four, and six-month diphtheria, tetanus, and pertussis (DTaP) vaccinations [3]. While there have been many case reports with a multitude of similar symptoms suggesting an association between the human papillomavirus vaccination causing postural orthostatic tachycardia syndrome (POTS) and dysautonomia (with some suggesting an immune-mediated autonomic dysfunction [4-5]), the American Autonomic Society published a position paper refuting any such link [6]. Hviid et al. found the temporal association possible but unlikely [7].

Known side effects from the Moderna and Pfizer/BioNTech COVID-19 vaccine include fever, fatigue, headache, myalgias, and arthralgias, usually within one to two days of vaccination (more commonly after the second dose). Other more rare adverse reactions include anaphylaxis, seventh cranial nerve palsy, and orofacial edema [8]. While every 1° Celsius elevation in core body temperature is associated with an increase in heart rate of about 10 beats per minute, it is highly unlikely that a subclinical temperature would cause tachycardia with heart rates over 130 beats per minute.

## Conclusions

Tachycardia is a nonspecific sign with a broad differential. Many side effects have been reported after the administration of vaccines. To our knowledge, this is the first reported case of the Pfizer-BioNTech COVID-19 vaccination being associated with tachycardia refractory to treatment with no other symptoms. Clinicians should be aware that this appears to be benign and, in our patient, resolved within 24 hours.
